# Shear Band Formation in Thin-Film Multilayer Columns Under Compressive Loading: A Mechanistic Study

**DOI:** 10.3390/ma18174215

**Published:** 2025-09-08

**Authors:** Yu-Lin Shen, Kasandra Escarcega Herrera

**Affiliations:** Department of Mechanical Engineering, University of New Mexico, Albuquerque, NM 87131, USA; kescarcega@unm.edu

**Keywords:** shear band, multilayers, pillar compression, finite element modeling

## Abstract

Micro-pillar compression is a popular experimental technique used for characterizing the mechanical behavior of nano- and micro-laminates. The compressive stress–strain response of the column-shaped thin-film composite can be measured, and the deformation and damage features can be revealed by post-test cross-section microscopy. The development of plastic instability in the form of localized strain concentration (shear bands), leading to eventual failure, is frequently observed. In the present study, a computational approach is used to illustrate the commonality of shear band formation from a continuum standpoint. Systematic finite element analyses are conducted, showing that the strain field tends to become localized once plastic yielding commences. Distinct shear offsets of the layered structure can be revealed from the numerical model, which is similar to those observed in experiments. The actual appearance of shear bands depends on the materials’ constitutive behavior and precise geometries. Post-yield strain hardening reduces the propensity of shear band formation, while strain softening enhances it. Imperfections such as the undulated layer geometry, as well as the frictional characteristics between the specimen and test apparatus, can also influence the shear band morphology and overall stress–strain response.

## 1. Introduction

Materials composed of alternating layers of thin films have received considerable attention, due to their novel or optimized performance in various structural and functional applications [1,2,3]. The multilayered structure may be metal/metal based (for achieving, e.g., ultrahigh strength or ductility enhancement) or metal/ceramic based (for achieving, e.g., improved toughness, wear resistance, and thermal stability). Their individual layer thickness is typically in the range of nanometers or micrometers. For instance, Al/Ni and Cu/Nb metallic nanolaminates have been studied for high-strength/lightweight [4] and radiation damage resistance [5] applications, respectively. Unique deformation characteristics were also found for Al/SiC metal/ceramic systems [6]. Ceramic nanolayers such as ZrO_2_/Al_2_O_3_ and HfO_2_/Al_2_O_3_ also find potential energy storage applications [7]. The metal/metal and metal/ceramic systems frequently have the alternating soft and hard combination, where the hard layers provide mechanical strength and the soft layers in between offer ductility for overall toughness enhancement. One popular experimental technique in characterizing the mechanical behavior of nano- and micro-laminates is micro-pillar compression [8,9,10,11,12,13,14,15]. The pillars with lateral size of a few hundred nanometers to several micrometers are typically prepared by focused ion beam (FIB) milling, and a flat-bottom indenter is used to press onto the column-like specimen. The compressive stress–strain response can thus be measured, and the deformation/damage features can be revealed by post-test cross-section microscopy. A common observation of deformed columns is the development of localized strain concentration (shear bands) during plastic deformation [16,17,18,19,20,21,22,23,24,25,26].

**Figure 1:**
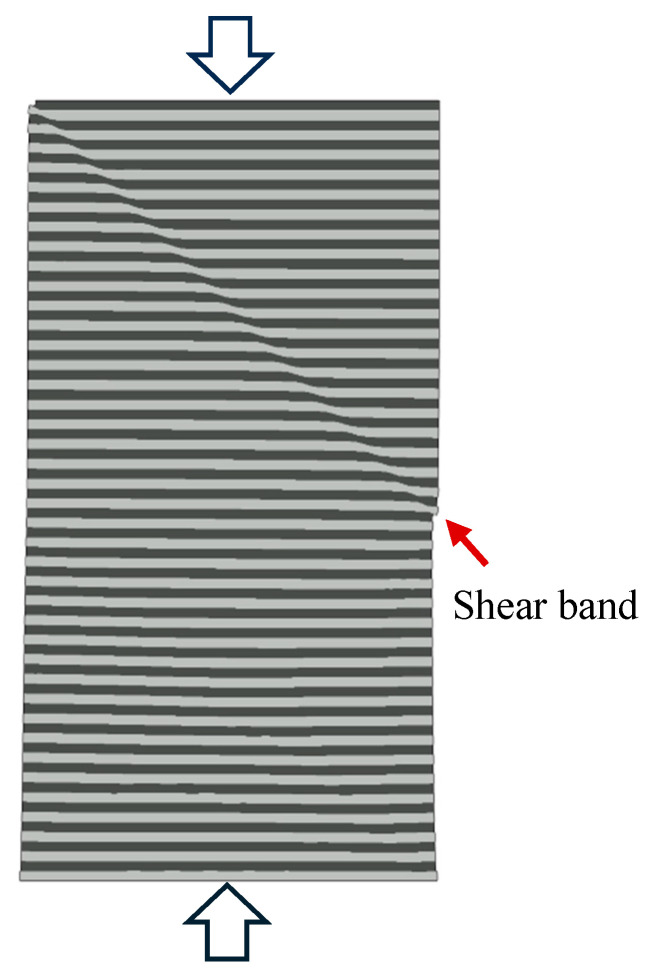
Deformed configuration of a multilayered column under compression obtained from numerical simulation. The darker and lighter regions represent the two alternating thin-film materials. A distinct shear band has developed, as commonly observed in pillar compression experiments.

Figure 1 shows a simulated image of shear band formation in a multilayered column consisting of alternating soft/hard layers (details are given below in this paper). While in actual experiments the exact shear-band configuration depends on the material, layer thickness, specimen geometry, loading condition, alignment of test device, and existence of any material and/or geometric imperfections, the simulated deformation in Figure 1 captures the essence of strain localization in experimental observations. Although the nominally planar layers are perpendicular to the compressive loading direction, the dominant shear path traverses through multiple layers at an inclined angle of approximately 45° which is the orientation of maximum shear. Once initiated, further deformation concentrates along these shear bands, leading to eventual fracture. Similar shear banding behavior is also frequently observed in multilayered thin films deformed by nanoindentation [27,28,29,30,31,32], although, under indentation, the deformation is highly nonuniform and not uniaxial, with the shear band angle also depending on the geometry of the indentation contact.

The initiation of strain localization is typically ascribed to nanoscale deformation mechanisms and their interaction with the high density of interfaces in thin-film laminates. Factors affecting shear band formation have been attributed to, for example, interfacial strength and morphology [33,34], interactions between interface with dislocations or deformation twins [16,35,36], grain boundary sliding and rotation [37,38], surface flaws [39], and amorphous/crystalline layer interaction [15,18]. In different material systems, shear banding may be facilitated by different physical mechanisms pertaining only to specific combinations of thin-film laminates. A more holistic approach to studying plastic instability is to utilize mechanics-based methodologies that may be applied to generic multilayer systems, which is the primary objective of the current work. Note that phenomenological continuum modeling has long been employed to study shear band formation in elastic-plastic solids [28,40,41,42,43]. In the present study, we attempt a computational approach to illustrate the commonality of shear band formation in alternating hard/soft layers from a pure continuum standpoint. For multilayered columns under compression, we show that mechanical strain can become easily localized once plastic yielding commences. Shear banding then takes over, unless other forms of deformation or damage become prevalent. The actual appearance of shear bands will depend on the materials’ constitutive behavior and precise geometries.

## 2. Numerical Model Description

**Figure 2:**
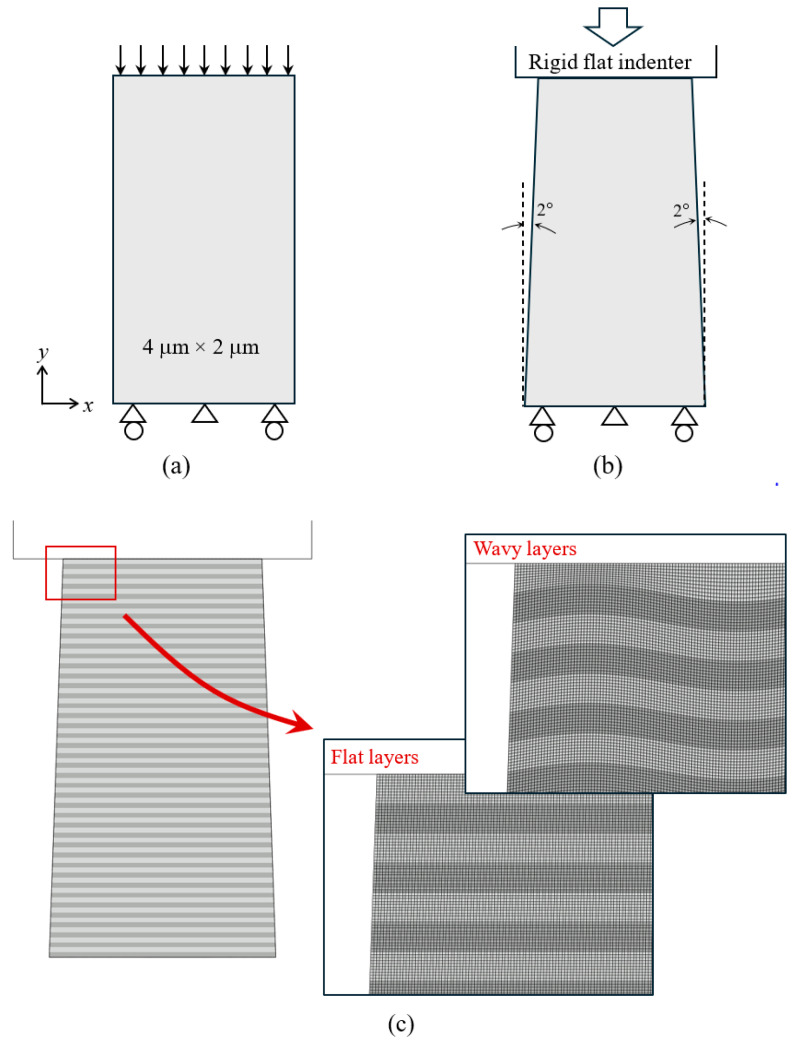
Schematics of (**a**) the uniform loading model and (**b**) the tapered column model, along with the loading and boundary conditions. (**c**) Tapered column with 40 hard layers and 40 soft layers, along with the finite element mesh for the flat-layer geometry and wavy-layer geometry. Four-noded plane strain elements are used throughout the simulation domain.

Figure 2a shows the reference numerical model along with the loading and boundary conditions. It is a 4 μm by 2 μm rectangular domain under uniform compression, with the displacement imposed on the top boundary. The y-displacement at the bottom boundary is prohibited while tangential slide is allowed, with the center point entirely fixed in x and y. To more accurately represent the micro-pillar compression test, the model shown in Figure 2b is utilized where a 2° taper exists (a tapered shape is commonly seen in actual materials fabricated by ion beam milling). For this geometry, the top of the column has a width of 2 μm and the height is 4 μm. Instead of prescribing the displacement on the top boundary, loading is implemented through contact between the specimen and a rigid flat indenter where the downward displacement is applied.

Figure 2c shows the multilayered structure with representative finite element meshes. A total of 40 “hard” layers and 40 “soft” layers are included, with the bottom and top layers being hard and soft, respectively. The mechanical properties used for the hard and soft materials are defined below. Each layer is 0.05 μm thick before deformation. To ensure mesh convergence, 10 rows of quadrilateral elements are included in each layer. A mesh convergence analysis is presented in Appendix A of this paper. Perfect bonding at the interfaces between layers is assumed. In most simulations, the initial layer geometry is flat. To study the effects of undulated geometry (frequently existent in nanolayers [6]), a wavy form of layers is also examined as shown in Figure 2c. The sinusoidal form features an amplitude of 0.015 μm and a wavelength of 0.5 μm. The top and bottom boundaries of the wavy models remain straight and horizontal.

**Figure 3:**
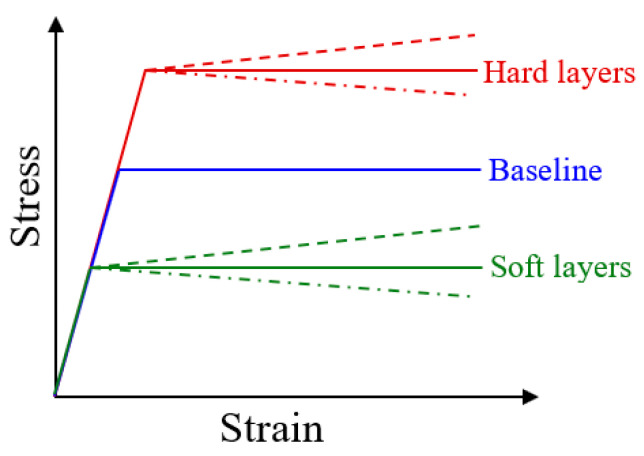
Schematics of the constitutive stress–strain behavior considered in the parametric study. A baseline elastic-perfectly plastic response serves as the reference material. The multilayers then consist of “hard” and “soft” layers with increased and decreased yield strengths, respectively. Possible linear strain hardening and strain softening are also considered.

The materials used in the finite element analyses are taken as isotropic elastic-plastic solids. The generic stress–strain behavior is schematically shown in Figure 3. Plastic yielding follows the von Mises criterion and incremental flow theory. An elastic-perfectly plastic homogeneous material serves as the baseline. Yield strengths higher and lower than the baseline value are then explored for the hard and soft layers, respectively. Additional considerations include the possible strain hardening and strain softening for both the hard and soft layers. The elastic properties are kept the same in all cases: Young’s modulus of 100 GPa and Poisson’s ratio of 0.3. The material models considered in this study are listed in Table 1. The baseline case “H700” is a homogeneous material with a constant yield strength (*σ*_y_, or plastic flow stress) of 700 MPa, chosen arbitrarily. Layered structures are designated by “L” with two numbers representing the yield strengths (in MPa) deviating equally from 700 MPa for the soft and hard layers, such as “L650/750” and “L600/800”. In order to cover a wider range of material parameters, additional models with a higher average yield strength (H1000 and L700/1300) and lower average yield strength (H400 and L100/700) are also included in the study, as listed in Table 1. The designations in Table 1, with additional letters “h” or “s”, represent cases with linear strain hardening or linear strain softening upon initial yielding. The hardening and softening slopes, measured as the rate change in stress with plastic strain (Δ*σ*/Δ*ε*^p^), are assumed to be +30 MPa and −30 MPa, respectively. Stronger rates of hardening and softening of ±60 MPa are also used to further demonstrate the generality of the simulation outcomes.

All simulations were carried out using the finite element software ABAQUS (version 2022) under the plane strain condition. Due to the need for adequately refined mesh in each layer of the multilayer structure, three-dimensional simulations will be extremely computationally demanding. While the current modeling is two-dimensional, it is useful for comparing qualitative trends resulting from different modeling parameters [44]; the evolution of shear bands can also be easily visualized. Unless otherwise stated, a coefficient of friction of 0.1 is assumed for the contact between the top layer and rigid indenter [8,27,28]. Quasi-static loading is implemented through an increasing applied displacement. In the presentation of numerical results, we focus on the appearance of shear bands, the overall engineering stress-strain response of each model, and the representative deformation patterns.

## 3. Numerical Results

### 3.1. Uniform Compression of Straight Columns

**Figure 4:**
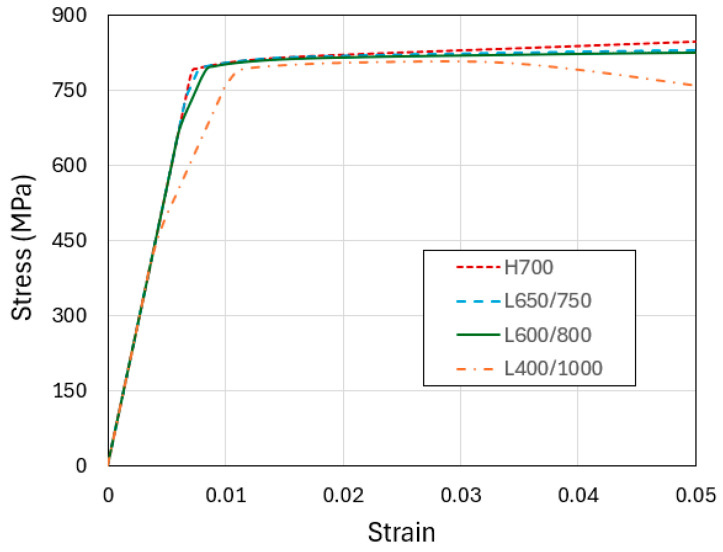
Engineering stress–strain curves obtained from the simulations of uniform compression of the four straight-column models: H700, L650/750, L600/700, and L400/1000.

We first consider the loading configuration in Figure 2a, where uniform compression is applied on the top face of a straight column (without taper). Four models are included: H700, L650/750, L600/800, and L400/1000. Figure 4 shows the simulated compressive engineering stress–strain curves of these models, up through the applied compressive engineering strain of 0.05. The distinct elastic-plastic feature is evident. Although the material is assumed to be perfectly plastic, the H700 curve displays a higher apparent yield stress due to the constraint of the plane strain condition. In addition, its post-yield portion shows a slight hardening trend because of the continuously increasing cross-section (leading to higher reaction force to maintain a constant true flow stress). For the multilayered models, the plastic flow stresses largely follow a decreasing trend from L650/750 to L600/800 and then L400/1000. With the explicit layers in the models, the softer material tends to absorb greater deformation, leading to a weakening effect which is most obvious for L400/1000 as the applied compressive strain becomes larger. It is also worth mentioning that, for L600/800 and L400/1000, the elastic portion of the curves shows two distinct apparent slopes. The first part is when both the soft and hard materials are elastic; in the second part the hard layers remain elastic while the soft layers have largely yielded. For L650/750, the difference in yield strength between the soft and hard layers is small so this two-stage process is less apparent.

**Figure 5:**
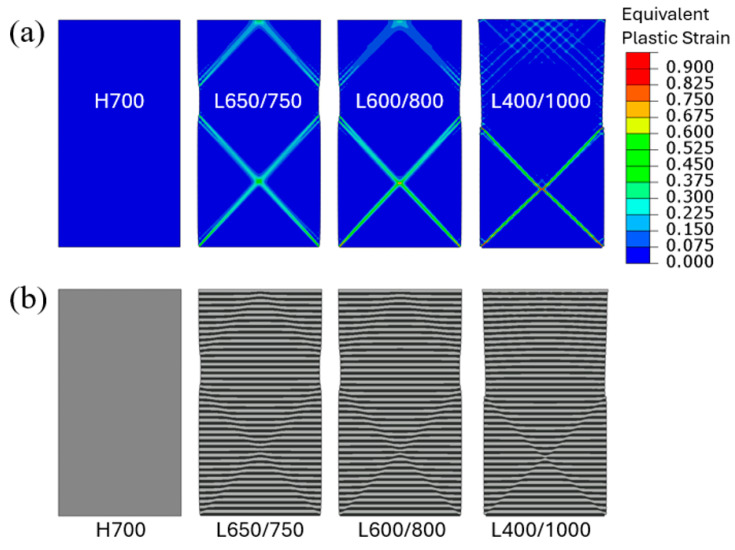
Uniform compressive loading of straight columns: (**a**) Contours of equivalent plastic strain and (**b**) deformed configurations, for the four material models when the applied engineering compressive strain is 0.04.

Figure 5a shows the contour plots of equivalent plastic strain when the applied engineering compressive strain is at 0.04 (well into the fully plastic regime as seen in Figure 4. The homogeneous model (H700) displays a uniform plastic strain field as expected (the actual plastic strain value is 0.0384). However, highly concentrated plastic strain patterns along the nominal maximum shear (45°) directions are observed in all multilayered models. For L650/750, the small difference in yield strength between the soft and hard layers is sufficient to trigger deformation localization. As the yield-strength disparity increases, strain concentration becomes more distinct with the local maximum equivalent plastic strain well exceeding 0.9 in the case of L400/1000 when the overall applied strain is 0.04. Figure 5b shows the deformed layer configurations under the same applied strain. The formation of shear bands along the deformation localization in the layered structure is evident, with the stronger plastic concentration leading to a greater soft/hard layer offset.

As demonstrated above, shear band formation can be predicted from the simulations in the explicit layered structure, while the deformation is uniform in their homogeneous counterpart. The origin of this deformation instability will be discussed in Section 4.

### 3.2. Flat-Indenter Compression of Tapered Columns

**Figure 6:**
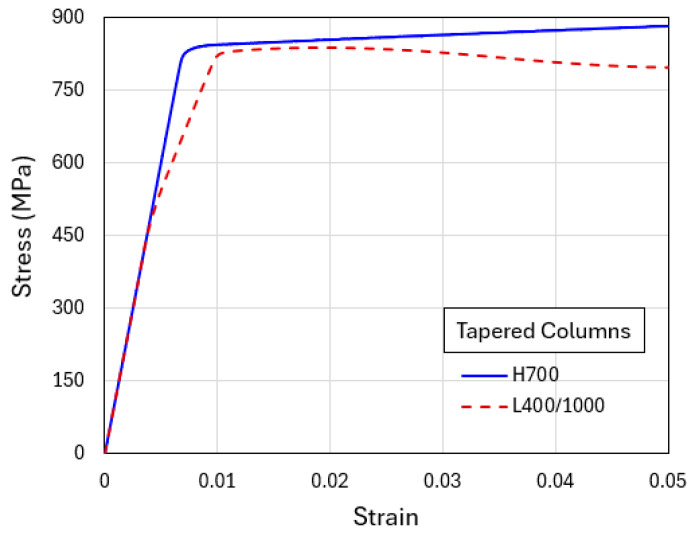
Engineering stress–strain curves obtained from the simulations of flat-indenter compression of tapered columns with material models of H700 and L400/1000.

The more realistic model configuration is now considered, where loading is applied through a rigid flat indenter pressing the specimen with a tapered geometry (Figure 2b). In the presentation, we focus on the two standard material models, H700 and L400/1000 (L650/750 and L600/800 resulted in similar features as L400/1000). Figure 6 shows the simulated compressive engineering stress–strain curves. As before, the homogenized H700 model displays a higher plastic flow stress than the model with explicit layers L400/1000.

**Figure 7:**
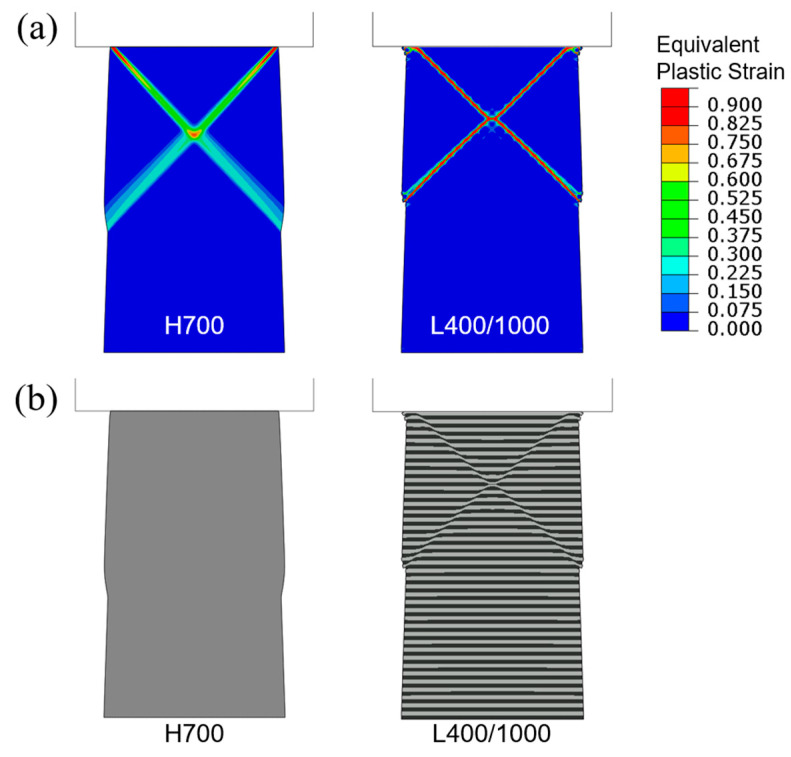
Flat-punch compression of tapered columns: (**a**) Contours of equivalent plastic strain and (**b**) deformed configurations, for H700 and L400/1000 when the applied engineering compressive strain is 0.04.

Figure 7a shows the contour plots of equivalent plastic strain in the tapered H700 and L400/1000 models, when the overall applied compressive strain is 0.04. The corresponding deformed configurations are in Figure 7b. Due to the narrower cross-section at the top, deformation is mostly carried by the upper portion where lateral bulging is apparent. One distinct feature is that the homogeneous material (H700) also displays concentrated plastic bands (which is not the case in H700 with a uniform initial width in Figure 5a). Compared with Figure 5a, the plastic strain concentration is significantly greater in the tapered structure in Figure 7a for both H700 and L400/1000. In Figure 7b, the layered geometry of L400/1000 also displays a pair of sharp shear bands with strong shear offsets. In this layered structure, at the current compression level, the shear offset brought about by the plastic instability has reached beyond one layer thickness.

**Figure 8:**
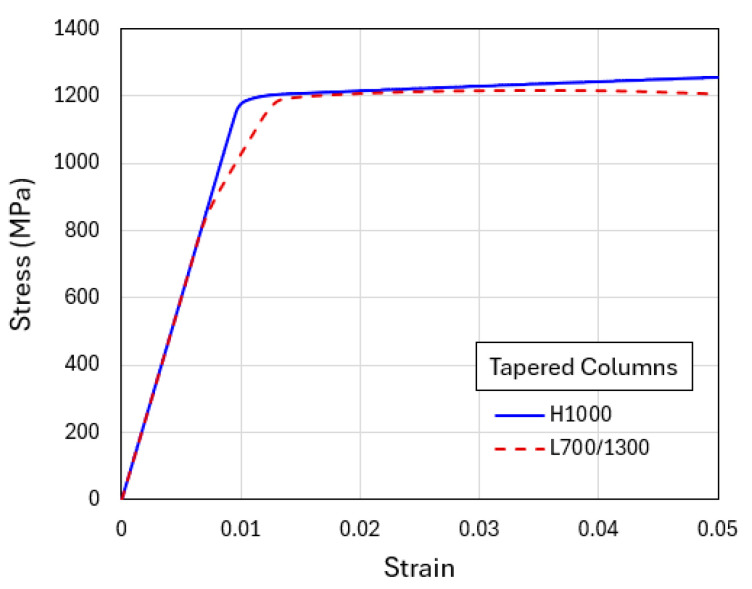
Engineering stress–strain curves obtained from the simulations of flat-indenter compression of tapered columns with material models of H1000 and L700/1300.

**Figure 9:**
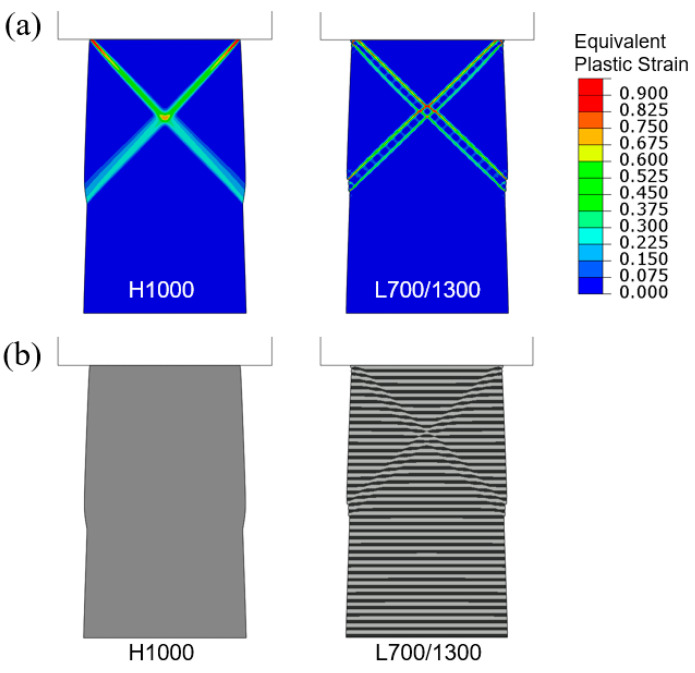
Flat-punch compression of tapered columns: (**a**) Contours of equivalent plastic strain and (**b**) deformed configurations, for H1000 and L700/1300 when the applied engineering compressive strain is 0.04.

**Figure 10:**
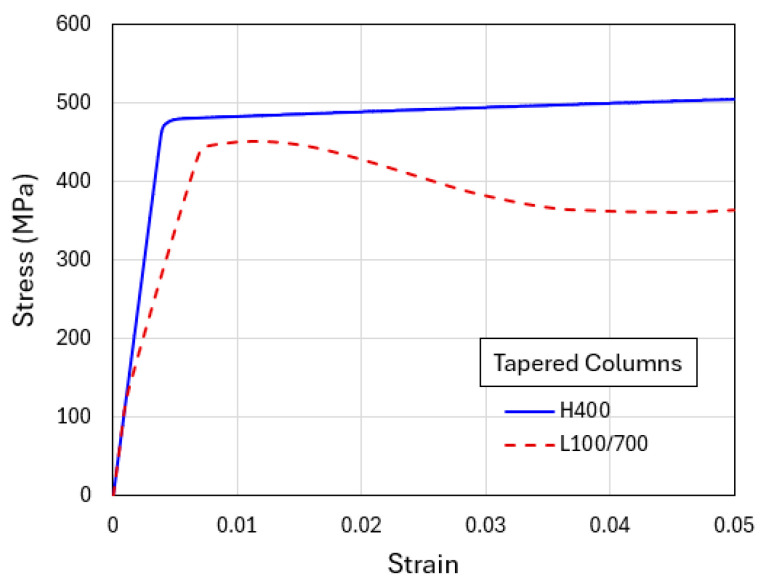
Engineering stress–strain curves obtained from the simulations of flat-indenter compression of tapered columns with material models of H400 and L100/700.

**Figure 11:**
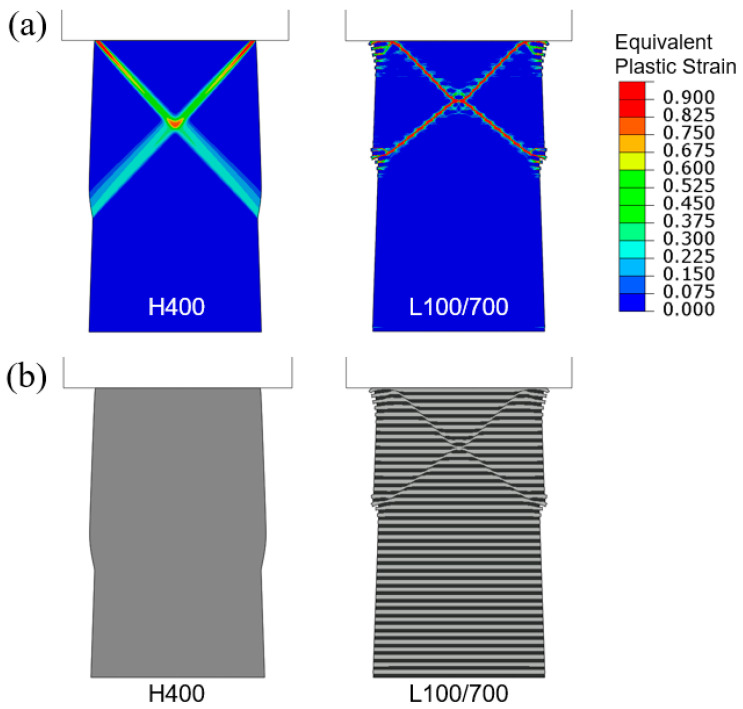
Flat-punch compression of tapered columns: (**a**) Contours of equivalent plastic strain and (**b**) deformed configurations, for H400 and L100/700 when the applied engineering compressive strain is 0.04.

To illustrate the generic shear banding features obtainable from the current modeling approach, two additional sets of results based on the higher average yield strength (1000 MPa) and lower average yield strength (400 MPa) are now presented. Figure 8 shows the simulated compressive engineering stress–strain curves for H1000 and L700/1300. The homogenized H1000 case results in a slightly higher plastic flow stress than L700/1300. Figure 9a shows the contour plots of equivalent plastic strain in the tapered H1000 and L700/1300 models, when the overall applied compressive strain is 0.04. The corresponding deformed configurations are in Figure 9b. It can be seen from Figure 8 that the difference between the two stress–strain curves is smaller than the standard case (H700 and L400/1000 shown in Figure 6), due to the overall higher strength which also leads to more diffuse shear bands observed in Figure 9. On the other hand, the weaker material models (H400 and L100/700) result in an opposite trend, as revealed by the corresponding stress–strain curves in Figure 10 and the equivalent plastic strain field and deformed configurations in Figure 11. The L100/700 stress–strain curve in Figure 10 is at a lower position compared to H400, and more distinct shear localization in L100/400 is evident in Figure 11. The shear steps and extruded soft layers are particularly notable in Figure 11. Comparing Figure 7, Figure 9 and Figure 11, the extent of shear offset displayed by the displaced layers follows an increasing trend from the stronger material (L700/1300) to the weaker material (L100/700), with the standard material (L400/1000) in between.

It is worth mentioning that the yield strength considered in this study spans a very wide range from the softest layer (100 MPa) to the hardest layer (1300 MPa). Many actual thin-film materials display their yield strengths within this range. For instance, the yield strength or plastic flow stress of Al and Cu thin films are around 100–400 MPa, depending on their thickness and microstructure [44], while those of Ti, Fe, and various other pure or alloyed metals can be much stronger and well above 1 GPa [3]. While different combinations showed different levels of strain concentration, all simulations led to shear band formation with similar qualitative features.

### 3.3. Effects of Strain Hardening and Softening

Continuing with tapered columns under flat-indenter loading, we now examine how post-yield strain hardening and strain softening may affect the shear band formation. Built upon the standard case of L400/1000, the last four material models defined in Table 1 are considered: L400 h/1000 h (soft and hard layers both undergoing strain hardening), L400 h/1000 s (soft layers undergoing strain hardening and hard layers undergoing strain softening), L400 s/1000 h (soft layers undergoing strain softening and hard layers undergoing strain hardening), and L400 s/1000 s (soft and hard layers both undergoing strain softening).

**Figure 12:**
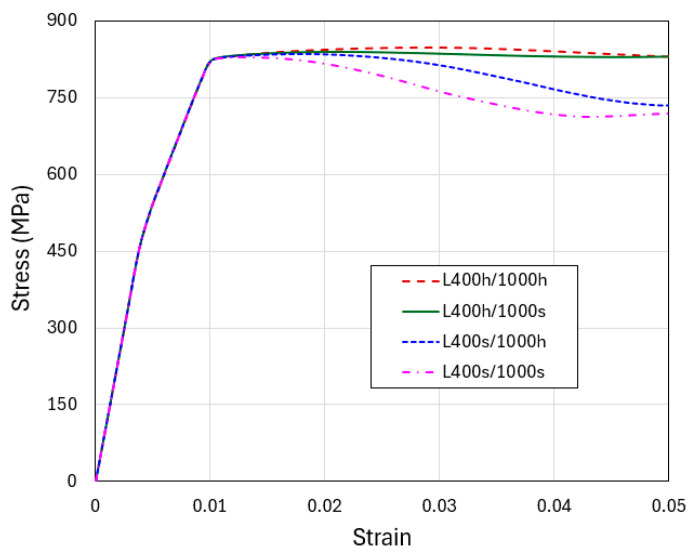
Engineering stress–strain curves obtained from the simulations of flat-indenter compression of tapered columns with material models of L400 h/1000 h, L400 h/1000 s, L400 s/1000 h, and L400 s/1000 s. The hardening and softening slopes are taken as Δσ/Δε^p^ = ±30 MPa.

Figure 12 shows the simulated compressive engineering stress–strain curves. In all four cases, the elastic responses up through initial yielding are identical. The combinations of strain hardening and softening lead to different post-yield behaviors. The strongest and weakest models are L400 h/1000 h and L400 s/1000 s, respectively, as expected. When one material can strain-harden and the other strain-softens, the soft-layer material generally plays a more important role since it tends to carry more of the post-yield deformation. As a consequence, the curve of L400 h/1000 s possesses a higher flow stress compared to L400 s/1000 h, especially at large, applied strains.

**Figure 13:**
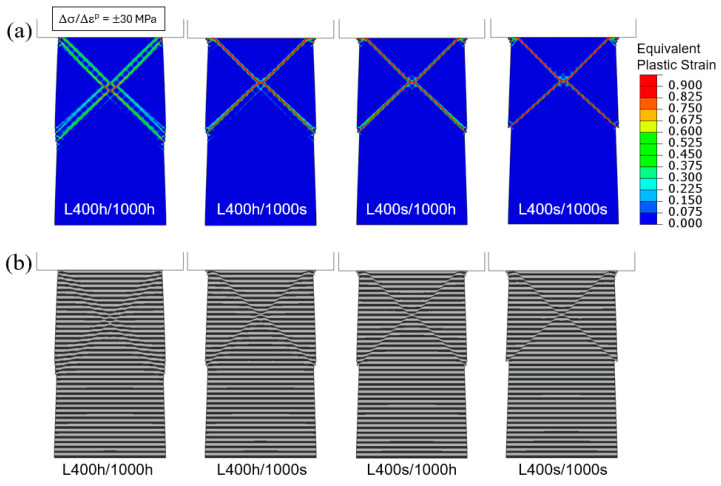
Flat-indenter compression of tapered columns: (**a**) Contours of equivalent plastic strain and (**b**) deformed configurations, for the four material models with strain hardening or softening when the applied engineering compressive strain is 0.04. The hardening and softening slopes are taken as Δσ/Δε^p^ = ±30 MPa.

The contour plots of equivalent plastic strain and the deformed layer configurations are shown in Figure 13. As before, these snapshots correspond to an overall compressive strain of 0.04. In all cases, the localization of plastic deformation is evident. However, if both the soft and hard layers are allowed to strain-harden, the plastic bands become more diffuse and the shear offsets are less conspicuous (L400 h/1000 h, the left-most images in Figure 13a,b). The extreme case is shown in the right-most images of the figure (L400 s/1000 s) where the soft and hard layers both strain-soften upon yielding. Here, the plastic bands are very sharp, with the greatest strain values among all cases, leading to very prominent shear offsets. The other two models, L400 h/1000 s and L400 s/1000 h, exhibit intermediate behavior, with the strain-softening soft layers (L400 s/1000 h) leading to more severe strain concentration between the two. In Appendix B, we use additional strain hardening and softening parameters to illustrate the generality of the observations described above.

## 4. Discussion

The current study numerically demonstrates that it is natural for plastic instability to occur during compressive testing of columns (pillars), and the multilayered geometry amplifies this localization effect. In actual experiments, the layered structure also makes shear bands readily visible microscopically. The simulations in the current study are solely based on the continuum-level analyses, without consideration of any microstructural or crystallographic mechanisms, grain boundary effects, and defect–interface interactions, etc. Therefore, the initial shear band orientation is along the nominal maximum shear direction of 45°. This is typically the angle observed in actual pillar compression experiments. Of course, as compressive deformation continues, the angle will appear shallower due simply to the geometric effect of vertical compression. Furthermore, the extent of shear offset, as revealed by microscopy, also depends on the overall applied compressive strain, so direct comparisons with the current modeling will be difficult. Nevertheless, the simulated shear bands resemble the cross-section microscopic images observed in many compression tests on micro-pillars with alternating thin-film layers [16,17,18,19,20,21,22,23,24,25,26]. In the current numerical work, post-yield strain hardening is found to reduce the propensity of shear band formation while strain softening promotes it. In actual materials, strain softening may be induced by internal damage such as microvoids or microcracks [40,43], by crystal lattice rotation causing easier crystallographic slip [45], or by thermal effect due to heat generation [41]. The phenomenological strain-softening approach has been used in modeling shear banding triggered by nanoindentation of thin-film laminates [28].

One limitation of the present approach is its two-dimensional nature under the idealized plane strain condition. The possible 3D effects, such as certain pre-existing flaw geometry, cannot be captured. Three-dimensional modeling will be left as future work, where different loading orientations, interface properties, various forms and distributions of defects, and thickness ratio, in conjunction with crystal plasticity and possible rate-dependent effect, can be built-into the finite element multilayer model. Extension to other geometric forms of dissimilar material joints may also be explored [46,47]. It is worth mentioning that the nominal shear action itself is two-dimensional in nature, and the plane strain condition is typically adopted in continuum-based analyses on shear banding [40,41,45]. Note also that analogy may be drawn between the shear localization simulated in the current study and the limit analysis in plasticity theory. The theorems of limit analysis provide bounds on the loads under which an elastic-perfectly plastic body reaches a critical state where large increases in plastic strain become possible with limited or no increase in load [48]. The current finite element modeling, on the complex geometry with multiple materials, directly generates the deformation history.

**Figure 14:**
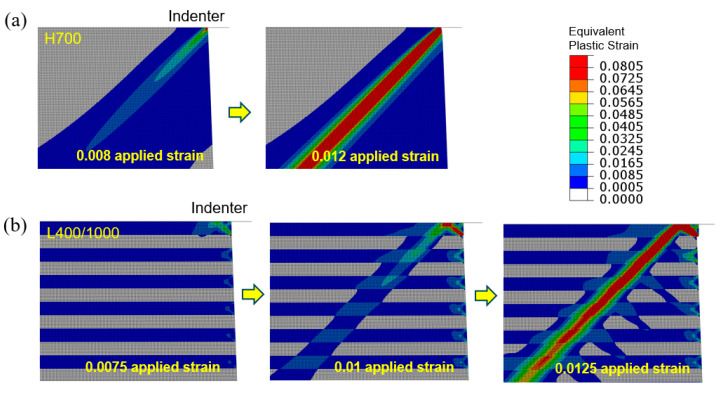
Evolution of equivalent plastic strain fields in the upper-right corner region, during flat-indenter compression of tapered (**a**) H700 and (**b**) L400/1000 columns. The applied engineering compressive strains corresponding to the snapshots are labeled.

The emergence of shear bands is frequently associated with material inhomogeneities giving rise to nonuniform deformation. In the present modeling scheme, the interface and corner regions act as inhomogeneities. Here, we examine the evolving plastic strain fields in tapered specimens of H700 and L400/1000 under flat-indenter compression as shown in Figure 14a,b, respectively. We focus on the region near the upper-right corner of the specimen (the upper-left corner region displays the same features with a symmetric pattern). The overall applied strain corresponding to each snapshot is also indicated. Note that these are the earlier stages leading to the deformation fields shown in Figure 7. In Figure 14a, local plastic deformation is initiated at the corner, due to the frictional slide of the top face and the minimum cross-section. Upon further compression, the concentrated plastic deformation extends in the 45° direction and develops into a band. It eventually crosses the one generated from the upper-left corner and reaches the free left-side surface, Figure 7a.

As for the layered structure shown in Figure 14b, under the 0.0075 applied compressive strain, the maximum local plastic strain appears at the lower-right corner of the top layer (softer material) adjacent to the hard layer underneath. This strong plasticity propagates along the 45° direction to the top face in contact with the indenter, and is seen to be “reflected” toward the lower-left direction and then traverses multiple layers when the applied strain attains 0.01. Significant shear displacing of the layers to create offsets generally starts at this stage. The shear band continues to grow in length and intensity, crossing the other one (originated from the upper-left region of the specimen) and reaching the free surface at the left side. In the layered structure the sharp shear steps at the free surface can be easily observed, Figure 7b.

**Figure 15:**
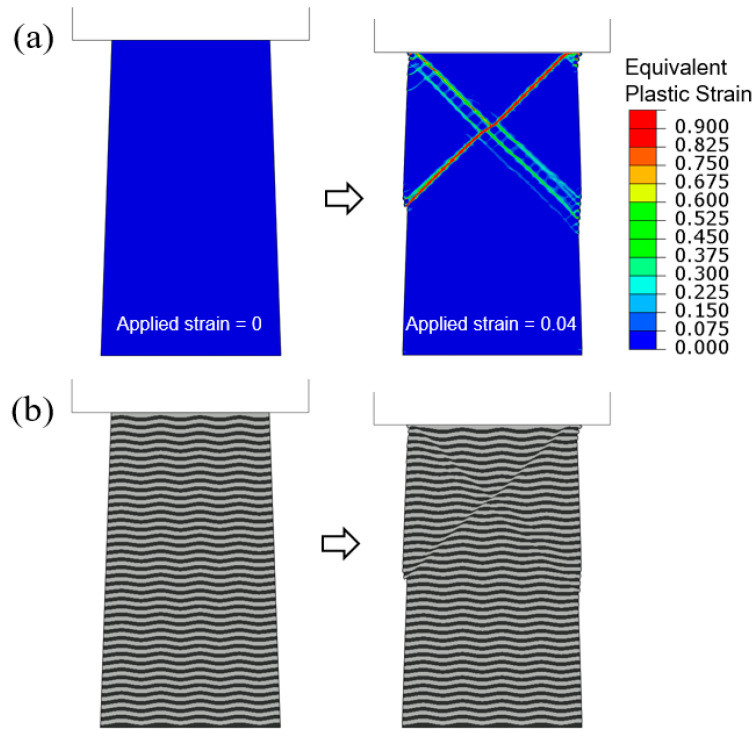
Flat-indenter compression of tapered L400/1000 column with undulated layer interfaces: (**a**) Contours of equivalent plastic strain and (**b**) deformed configurations, when the applied engineering compressive strain increases from 0 to 0.04.

In Figure 5, Figure 7, Figure 9, Figure 11 and Figure 13, the shear bands all develop in pairs and make an “X” shape. This is due to the perfect geometry along with symmetry used in the simulations. In actual pillar compression experiments, however, one dominant band along a single direction was frequently observed [16,19,20,22,23,25]. Here, we consider a numerical model with imperfect geometry, where the interfaces between layers take the undulated form, as defined in Section 2 and shown in Figure 2c. Flat-indenter compression is imposed on this tapered column with L400/1000. The contour plots of equivalent plastic strain and the overall multilayer configurations are shown in Figure 15a,b, respectively, when the applied compressive strain increases from 0 to 0.04. With the built-in wavy layers, the structure loses its symmetry about the central vertical axis. It is seen that one shear band from the upper-right to lower-left is now dominant, and the other becomes more diffuse. In Figure 15b, the shear step created at the left free surface is much more significant.

There are other ways to generate a single dominant shear band in the numerical model. The image in Figure 1 is, in fact, an example using the uniform compression scheme. Here, a defect was intentionally placed in the model by endowing one soft-layer element at the free side surface with the properties of the hard material. Note that in actual laboratory specimens created from thin-film laminates, various types of imperfections on the surface or inside the material can exist, which can dictate the shear band configuration. As in Figure 15b, this type of single dominant band emerging from the top contact face is frequently observed in multilayer pillar compression experiments [16,19,20,22,23,25].

**Figure 16:**
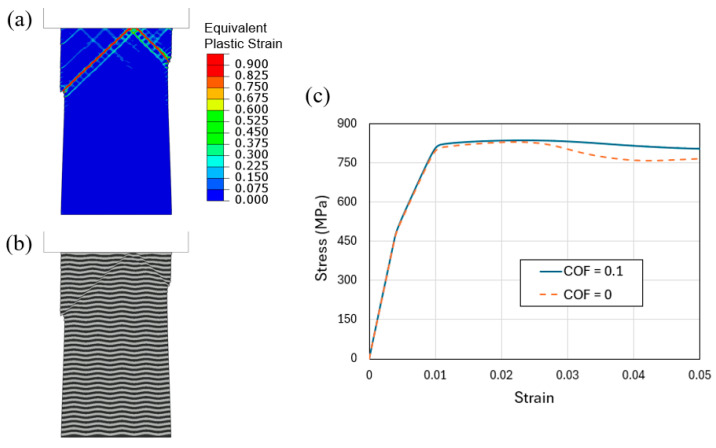
Flat-indenter compression of tapered L400/1000 column with undulated layer interfaces, if free tangential slide is allowed between the top face and the indenter (i.e., coefficient of friction is zero instead of 0.1): (**a**) Contours of equivalent plastic strain and (**b**) deformed configurations, when the applied engineering compressive strain increases from 0 to 0.04. (**c**) Overall stress–strain curves of the flat-indenter compressed tapered columns with undulated layers, with two different coefficients of friction (COF) of 0 and 0.1.

Another potentially important factor in affecting the shear band formation is the frictional characteristics at the contact between the top of the specimen and the indenter. In all the simulations presented above, the coefficient of friction (COF) is taken as 0.1. Here, we consider one case of undulated layer geometry allowing free tangential slide at the top face, i.e., COF = 0. The contour plot of equivalent plastic strain and the deformed multilayer configuration, when the applied strain is 0.04, are shown in Figure 16a,b, respectively. There are two primary shear bands generated. However, the pattern is not symmetrical. From Figure 16a, it can be seen that multiple sets of localized plastic bands have developed (due to the periodic peak/valley wavy geometry and free tangential movement at the contact), but eventually two major bands carry most of the deformation. The shear step at the left surface caused by the longer band is particularly distinct, as in Figure 16b. It is worth mentioning that this type of unsymmetric shear band emerging from the top contact has been observed in compressed Al/Pd [17] and Cu/PdSi [18] nanolayer pillars.

Figure 16c shows the simulated overall compressive engineering stress–strain curves of the flat-indenter compressed tapered columns with wavy layers, with two COFs of 0 and 0.1. The different frictional properties lead to different stress–strain behavior, with the greater COF giving rise to a higher plastic flow stress. This finding illustrates the fact that, in interpreting and comparing experimental stress–strain responses, one needs to exercise caution because the measurement can be influenced by the actual frictional characteristics. This is in addition to any potential effect caused by the actual specimen geometry and pre-existing defects.

## 5. Conclusions

Numerical finite element analyses were carried out to study shear band formation in multilayered columns under compressive loading. The simulations utilized structures with a homogeneous domain as well as explicit layers with alternating soft and hard thin films, to represent typical micro-pillar compression experiments. The primary contributions of this work, beyond the traditional continuum-level approaches, are the incorporation of multilayer configuration (a wide range of elastic-plastic parameters for the hard/soft layers, tapered column, and mechanical contact with the indenter, etc.), to systematically examine the overall deformation and evolution of shear band morphology. Salient findings are summarized below:

- There is a tendency for plastic instability to occur during compressive testing of columns (pillars), and the multilayered geometry amplifies the localization effect. A smaller difference in yield strength between the hard and soft layers will reduce the tendency for shear banding-induced failure initiation.
- Instability-induced shear banding can be obtained from the phenomenological elastic-plastic numerical modeling, without consideration of any microstructural or crystallographic processes.
- Post-yield strain hardening reduces the propensity of shear band formation, while strain softening promotes it. Adopting thin-film layers with a stronger strain hardening ability will thus delay shear band-induced failure initiation.
- Imperfections, such as the undulated layer geometry, can influence shear band morphology.
- The overall compressive stress–strain curve is affected by the frictional characteristics of the contact between the top face (specimen) and flat indenter (testing apparatus). Care should be taken in interpreting and comparing experimental pillar compression results.
- Future studies using a similar modeling framework can include the effects of layer thickness ratio, rate- and/or temperature-dependent constitutive behavior, interfacial compliance and damage, and crystal plasticity.

## Figures and Tables

**Table 1:** List of various material models used in this study.

Model Designation	Initial Yield Strength (σ_y_)	Strain Hardening (Δσ/Δε^p^)
Homogeneous H700	700 MPa	0
L650/750	Soft layers 650 MPa	0
	Hard layers 750 MPa	0
L600/800	Soft layers 600 MPa	0
	Hard layers 800 MPa	0
L400/1000	Soft layers 400 MPa	0
	Hard layers 1000 MPa	0
Homogeneous H1000	1000 MPa	0
L700/1300	Soft layers 700 MPa	0
	Hard layers 1300 MPa	0
Homogeneous H400	400 MPa	0
L100/700	Soft layers 100 MPa	0
	Hard layers 700 MPa	0
L400 h/1000 h	Soft layers 400 MPa	+30 MPa
	Hard layers 1000 MPa	+30 MPa
L400 h/1000 s	Soft layers 400 MPa	+30 MPa
	Hard layers 1000 MPa	−30 MPa
L400 s/1000 h	Soft layers 400 MPa	−30 MPa
	Hard layers 1000 MPa	+30 MPa
L400 s/1000 s	Soft layers 400 MPa	−30 MPa
	Hard layers 1000 MPa	−30 MPa

**Figure A1:**
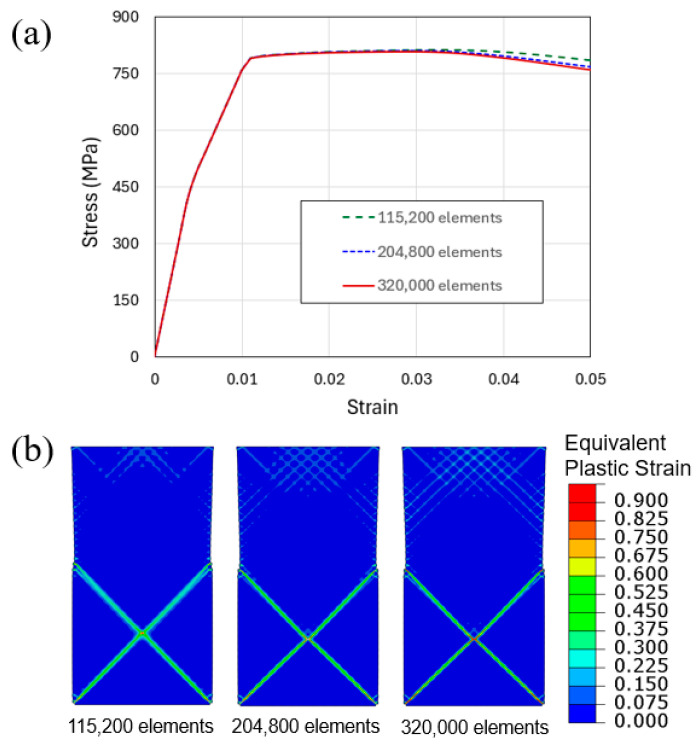
Mesh convergence analysis using the uniform compressive loading of straight columns of L400/1000 model: (**a**) Simulated compressive engineering stress–strain curves; and (**b**) contours of equivalent plastic strain when the applied engineering compressive strain is 0.04. Three levels of mesh densities, with a total of 115,200, 204,800, and 320,000 elements, are included.

**Figure A2:**
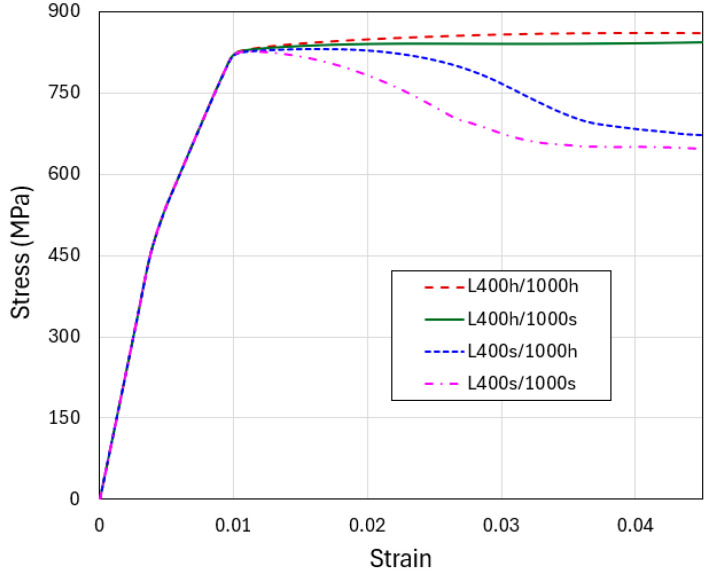
Engineering stress–strain curves obtained from the simulations of flat-indenter compression of tapered columns with material models of L400 h/1000 h, L400 h/1000 s, L400 s/1000 h, and L400 s/1000 s. The hardening and softening slopes are taken as Δσ/Δε^p^ = ±60 MPa.

**Figure A3:**
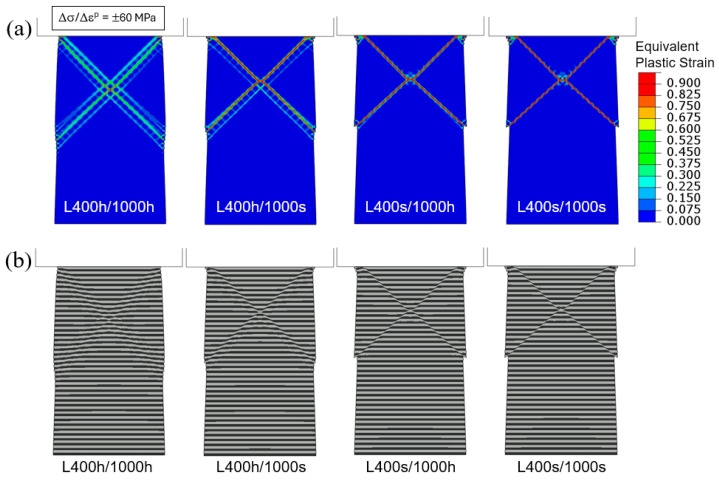
Flat-indenter compression of tapered columns: (**a**) Contours of equivalent plastic strain and (**b**) deformed configurations, for the four material models with strain hardening or softening when the applied engineering compressive strain is 0.04. The hardening and softening slopes are taken as Δσ/Δε^p^ = ±60 MPa.

## Data Availability

The original contributions presented in this study are included in the article. Further inquiries can be directed to the corresponding author.

## Appendix A

In this section, we present a convergence study of the finite element mesh, using the uniform compression model of L400/1000 as an example. Three levels of mesh density, with a total of 115,200, 204,800, and 320,000 quadrilateral linear elements, were utilized. Figure A1a shows the simulated engineering stress–strain curves. The equivalent plastic strain contours demonstrating the unique banded structure are shown in Figure A1b. It can be seen that the mesh densities give rise to nearly identical elastic and early yielding response; the plastic flow stress starts to display apparent difference when the applied compressive strain is greater than about 0.03. However, the two finer meshes lead to much closer flow behavior (<1% difference in flow stress) compared to the coarsest mesh. Figure A1b also demonstrates the same shear band features for the two finer meshes. The finest mesh (320,000 total elements) is thus used to generate all numerical results presented in this paper.

## Appendix B

The effects of post-yield strain hardening and strain softening were analyzed in Section 3.3. Here, we present additional simulations using a stronger rate of hardening and softening, ±60 MPa, applied to the four models of L400 h/1000 h, L400 h/1000 s, L400 s/1000 h, and L400 s/1000 s. Figure A2 shows the simulated compressive engineering stress–strain curves. The overall trend is qualitatively the same as that in Figure 12. The strongest and weakest responses are L400 h/1000 h and L400 s/1000 s, respectively. The L400 h/1000 s curve is higher than L400 s/1000 h, which is consistent with the case of hardening/softening rate of ±30 MPa as discussed in Section 3.3.

The contour plots of equivalent plastic strain and the deformed layer configurations are shown in Figure A3, with the snapshots corresponding to an overall compressive strain of 0.04 as before. The left-most images (L400 h/1000 h) show more diffuse plastic bands while the other extreme are shown in the right-most images (L400 s/1000 s), where very sharp shear offsets (greater than one layer thickness) are apparent. All qualitative features are the same as in Figure 13. The additional simulation results presented here further support the findings on the effects of strain hardening and softening reported in this study.
